# Editorial: New insights in small fruit diseases

**DOI:** 10.3389/fpls.2023.1306301

**Published:** 2023-11-08

**Authors:** Darko Jevremović, Irena Mavrič Pleško

**Affiliations:** ^1^ Department for Fruit Protection and Certification of Planting Material, Fruit Research Institute, Čačak, Serbia; ^2^ Plant Protection Department, Agricultural Institute of Slovenia, Ljubljana, Slovenia

**Keywords:** small fruits, diseases, fungi, phytoplasma, sustainable plant protection

Small or berry fruits, known as “superfoods”, are grown on all continents. They play an important role in human nutrition and health due to the nutritional characteristics and bioactive components of their fruits. They are also hosts of a wide range of pathogens that threaten their production. In susceptible species and cultivars, pathogens reduce yield and fruit quality, and occasionally they can even destroy entire orchards. This Frontiers Research Topic comprises five contributions from researchers exploring various aspects of the plant pathology of berry fruits.

In today’s agriculture, it is essential to decrease the use of synthetic pesticides for disease management in order to preserve the environment and human health. Many investigations are being undertaken to determine the efficacy of natural pesticides produced by plants against pathogens and pests. Gray mold is an economically important disease of numerous berry fruits caused by the fungal pathogen *Botrytis cinerea*. In recent years, many aromatic oils and plant extracts have been evaluated in the field of sustainable plant protection (Raveau et al., 2020). Antifungal substances, as an alternative to fungicides, can be used for the control of pathogens of economic relevance (such as *B. cinerea*). Dene et al. studied the effect of coriander (*Coriandrum sativum*) seed extracts and essential oils on grey mold. They showed that extracts of coriander seeds expanded the photosynthetic capacity and antioxidant reaction of strawberry plants but had a negative effect on gray mold suppression. The antioxidant response of strawberry plants was, in most cases, activated more by extract than essential oil. The results of the study showed that higher photosynthetic capacity values did not increase the antifungal effect following treatment with essential oils. A phytotoxic impact occurred when higher concentrations of the extract and essential oil of coriander seeds were used.

As already stated, the fungus *B. cinerea* triggers devastating yield losses in many different plants, including strawberries (*Fragaria vesca* L.). Current management approaches rely on the application of fungicides before harvest, which is restricted by rigorous legislation. β-aminobutyric acid (BABA), a defensive elicitor, has been shown to produce resistance against many pathogens, including *B. cinerea*, in a variety of agricultural plants. According to reports, BABA protects a variety of plant species against many pests and pathogens by activating defense pathways dependent or not on salicylic acid (Cohen et al., 2016; Wilkinson et al., 2018). BABA is produced by stressed plants and can also be synthesized chemically. To identify resistance effects of BABA Badmi et al. evaluated how directly applied BABA, soil-drenched BABA, and untreated strawberry plants responded to *B. cinerea* infection. Local application of BABA to leaves boosted disease resistance, while systemic application as a soil drench reduced plant growth and increased vulnerability to *B. cinerea* infection. According to this research, different studied factors (e.g. the tissue type, plant developmental stage, application method, and genotype) can impact the effect of defense elicitors on plant resistance. The findings of a study on *Botrytis cinerea* control in strawberries using plant extracts and plant amino acids have highlighted the significance of this field of study and emphasized the need for more research to fully implement successful application and treatment techniques.

*Phytophthora rubi* is a devastating pathogen causing the decay of raspberries (*Rubus idaeus* L.) in numerous countries throughout the world. Raspberry growers in North America are particularly concerned about Phytophthora root rot root disease (Sapkota et al., 2022). In order to comprehend the genetic mechanisms underpinning plant host adaptability, Tabima et al. (2017) reported the full genome of a *P. rubi* isolate originating from the Netherlands. In their current research, Sapkota et al. sequenced an additional 25 P*. rubi* isolates from various locations and different cultivars in Canada. According to the research, substantial genotypic diversity and limited gene diversity were found among sequenced isolates. The analysis revealed significant genotypic diversity and low gene diversity among sequenced isolates. This in-depth investigation has revealed new information and insight into the *P. rubi* genome structure, and it serves as an important global resource for future population diversity studies on this pathogen. Obtained results could also be useful for raspberry breeding programs to develop *Phytophthora*-resistant cultivars.

The highbush blueberry (*Vaccinium corymbosum* L.) and cranberry (*V. macrocarpon* Ait.) are native to North America but are grown all over the world in production orchards. Blueberry and cranberry yields in orchards can occasionally decrease over time, and the problem reappears even after replanting. In such a scenario, the main reason for reduced productivity is very likely linked to the soil. The diversity of rhizosphere microbiomes (bacteria and fungi) is regarded as a significant predictor of soil health (Schloter et al., 2018). The rhizosphere microbiome of cranberry and blueberry crops in New Jersey, USA, was studied by Kawash et al. 2023. When comparing blueberry and cranberry at the phylum level, little variations were observed in the composition of the bacterial microbiome, with Proteobacteria and Acidobacteria being the predominant ones. The fungal communities associated with blueberry and cranberry were quite different, dominated by the phyla Ascomycota and Basidiomycota. These findings establish a framework for investigating microbiomes that could affect the health of *Vaccinium* species in the Northeast USA.

Phytoplasmas are phloem-limited plant pathogenic bacteria associated with numerous diseases in plants. They induce a wide range of growth irregularities that can affect a plant’s appearance and negatively impact fruit production. It is still unclear how phytoplasma affects the host plant’s physiological functioning (Namba, 2019). In order to determine whether the response to phytoplasma infection is associated with variations in genome-wide DNA methylation, Liu et al. studied the methylome and transcriptome patterns of phytoplasma-infected mulberry plants. The authors identified a significant number of genes with varying levels of methylation and expression, and confirmed that phytoplasma infection can positively regulate plant disease resistance through methylation and expression of genes (in this case the Mu-GsSRK gene). The research provided new information about the mulberry-phytoplasma association.

When proposed, the aim of this Research Topic was to present the latest research and perspectives on berry fruit diseases. The research presented was conducted in different countries and continents, confirming the severity and distribution of the pathogens causing more or less detrimental diseases in small fruits. The funding data for the published manuscripts showed that all reported studies were financially supported by governmental and regional resources.

## Author contributions

DJ: Writing – original draft. IM: Writing – review & editing.
